# Evaluation of Radiogenomics for Risk Stratification of Intracranial Aneurysms: A Pilot Study

**DOI:** 10.21203/rs.3.rs-6674755/v1

**Published:** 2025-05-27

**Authors:** Sricharan S Veeturi, Kerry E Poppenberg, Nandor K Pinter, Vinay Jaikumar, Elad I Levy, Adnan H Siddiqui, Vincent M Tutino

**Affiliations:** University at Buffalo, State University of New York; University at Buffalo, State University of New York; University at Buffalo, State University of New York; University at Buffalo, State University of New York; University at Buffalo, State University of New York; University at Buffalo, State University of New York; University at Buffalo, State University of New York

## Abstract

Aneurysm wall enhancement (AWE) has emerged as an imaging biomarker, which could help in risk stratification of intracranial aneurysms (IAs) and also shed light on local pathobiology of the IA wall. In this pilot study, we explored the potential of a radiogenomics approach by combining blood-based biomarkers and AWE for better risk stratification of IAs. Patient specific vessel wall imaging scans and whole blood samples were obtained, and IAs were classified as high-risk or low-risk using two different metrics: symptomatic status (3 symptomatic vs. 13 asymptomatic) and PHASES score (4 with a high score vs. 12 with a low score). Radiomics features (RFs) were extracted from the pre- and post-contrast MRI for all IA sac walls, and significantly different RFs were identified through univariate analysis. RNA sequencing from whole blood samples for these patients was also performed to identify differentially expressed genes (DEGs) between high and low-risk IA groups. Using both risk metrics, we performed principal component analysis (PCA), clustering analysis, and correlation analysis between RFs and genes. Lastly, ontological analyses were carried out to investigate biological mechanisms associated with the DEGs. Our analysis of 16 IAs identified 22 RFs that were significantly different between symptomatic and asymptomatic IAs and 97 genes with at least a 2-fold change and a p-value < 0.01. Examining risk with respect to PHASES score, we identified 10 significant radiomics features and 38 differentially expressed genes. Furthermore, we found a significant correlation between 15 unique RFs and 49 DEGs. Through principal component analysis, we found that DEGs only and radiogenomics features produced a better separation between high- and low-risk, for both risk metrics, than RFs alone. We demonstrated that a radiogenomics approach can help in better risk stratification of IAs.

## Introduction

Intracranial aneurysms (IAs) are cerebral outpouchings that have high mortality and morbidity rates if they rupture^1,2^. Accurate and timely risk stratification is paramount for management of IAs^3^. Current size-based risk assessment of IA is not ideal as 50% of all ruptured IAs are < 5mm.^4^ Recently, aneurysm wall enhancement (AWE) has emerged as a potential tool to identify high-risk IAs^5,6^. Indeed, studies have shown that IAs exhibiting AWE have a higher degree of local wall inflammation, presence of vasa vasorum and neovascularization, which are indicative of IA instability and rupture^7–9^. However, longitudinal studies have shown that although AWE has a high sensitivity in identifying growing IAs, it has low positive predictive value^10^. A recent meta-analysis by Larson et al. has shown that AWE has a negative predictive value of 96% and a positive predictive value of just 14.4%^11^. Hence, combining AWE with biomarkers of different modalities can help improve its performance in risk stratification.

“Radiogenomics” is a potential approach to improving diagnostic performance of biomarkers by combining radiomics and genomics^12^. The core philosophy of radiogenomics is to fuse genomics that can give molecular level information, with image-based radiomics that reflect local pathophysiological manifestation of the disease^13^. Recent studies have implemented radiogenomics pipelines for disease prognosis and risk stratification particularly in oncological studies. For example, a study by Chaddad et al., reported a higher performance when using a radiogenomics approach for prediction of outcome of IDH1 wild-type glioblastoma patients^14^. Similarly, another study by Zeng et al., used a radiogenomics approach to predict overall survival of clear cell renal cell carcinoma where they observed that the predictive performance of the multi-omics model improved as compared to traditional radiomics model^15^. These studies underscore the utility of using a radiogenomics approach as compared to a unimodal analysis for clinical risk stratification.

In this study, we aimed to explore the potential utility of radiogenomics in risk stratification of a small cohort of IAs. To this end, we combined radiomics features obtained from a previously developed pipeline for AWE quantification with transcriptomic data obtained through whole blood RNA sequencing. Two different risk metrics were used to define high risk IAs. We then evaluated the performance of radiomics alone, genomics alone, and their combination in identifying high-risk IAs.

## Methods

### Patient Population and Aneurysm Characteristics

This study was approved by the Human Research Institutional Review Board at the University at Buffalo (STUDY030474433). Blood samples and vessel wall MRI scans were collected from patients undergoing angiographic imaging at Gates Vascular Institute (Buffalo, NY) between 2021 and 2023 as described previously^16^. Only IAs larger than 2 mm in size that had both pre- and post-contrast imaging were included in the analysis. We further excluded aneurysms that exhibited acquisition artifacts or were located in the cavernous segment of the internal carotid artery (ICA)^17^. In patients with multiple IAs, only the symptomatic IA as noted in the electronic medical record was included for further analysis. Demographics and comorbidities were obtained from the patients’ medical records. IAs were categorized into high-risk or low-risk aneurysms in two ways: 1) based on the symptoms of the patient at the time of blood collection, and 2) based on the PHASES score. Patients exhibiting severe headache or blurry vision within two weeks from presentation were classified as symptomatic patients^18^. Additionally, we computed PHASES for each IA as a secondary risk metric, and IAs having a PHASES ≥ 6 were classified as high-risk IAs^19^.

### Image Acquisition and Radiomics

Patient specific vessel wall imaging was done as described previously.^20^ Briefly, a 3T MRI scanner (Ingenia Elition 3.0 X, Philips healthcare) was used to acquire a time-of-flight, pre-contrast 3D T1, and post-contrast 3D T1 scans five minutes after the administration of contrast gadolinium (Gadobutrol, 0.1 mL/kg). Anonymized DICOM files were then imported into 3D Slicer to generate 3D segmentations of the IA wall.^6^ Radiomics features were extracted from the aneurysm wall in T1-pre and T1-post-contrast using pyradiomics package as previously described^6,21^. Extracted RFs included shape-based features, first-order features, and textural features.^22^ Additionally, difference between the first order features from the pre- and post-contrast MRI were also computed to use as features. A total of 293 RFs per case were extracted.

### RNA Sequencing and Analysis

**Figure 1:**
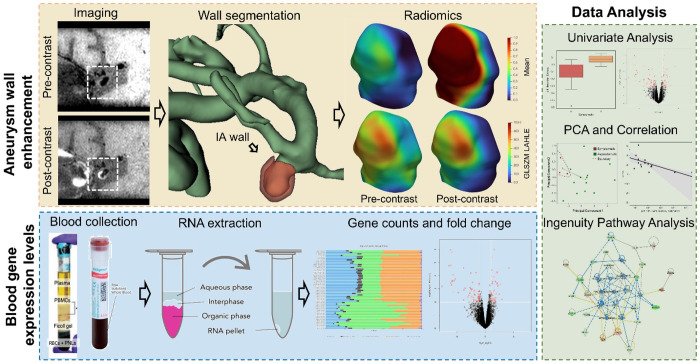
Workflow for analysis of AWE and RNA sequencing. Pre- and post-contrast MRI are registered onto each other, and we extracted radiomics. We also collected blood samples from the same patients and performed gene expression analysis after RNA extraction. We used both these modalities to study differences in radiomics, genomics, and radiogenomics in high and low-risk aneurysms.

For sequencing, RNA libraries were prepared using the Illumina TruSeq stranded total RNA gold kit (Illumina, San Diego, CA). All samples underwent RNAseq on the Illumina NovaSeq6000 or the HiSeq2500 System (Illumina) in a series of two batches. Samples were demultiplexed with Bcl2Fastq. Per-cycle basecall files generated by the NovaSeq6000 were converted to pre-read FASTQ files using bclfastq version 2.20.0.422 using default parameters. The quality of the sequencing was reviewed using FastQC v.0.11.5. Potential contamination detected using FastQ Screen v.0.11.1. Genomic alignments were performed using HISAT2 v.2.1.0 using default parameters. National Center for Biotechnology Information (NCBI) reference GRCh38 was used for the reference genome and gene annotation set. Sequence alignments were compressed and sorted into binary alignment map files using samtools v.1.3. Mapped reads for genomic features were counted using Subread featureCounts v.1.6.2 using the parameters ‘-s’ 2, ‘-g’ gene_id, ‘-t’ exon, ‘-Q’ 60; the annotation file specified with ‘-a’ was the NCBI GRCh38 reference from Illumina iGenomes. The entire pipeline for the current study is shown in Figure 1.

### Radiogenomics Analysis

For imaging features, we performed univariate analysis to identify significantly different RFs between high-risk and low-risk IAs, using the symptomatic status and PHASES score. RFs were evaluated for normality using Shapiro-Wilk test. Normally distributed RFs were evaluated using the Student’s *t*-test, and non-normally distributed RFs were tested using the Mann-Whitney U test. All the statistical analysis was performed using SciPy package in python^23^.

For gene expression features, we conducted differential expression analysis of the RNA sequencing data using R. We filtered dataset to transcripts with expression>0 in at least 50% of the samples and those corresponding to protein coding genes. This reduced dataset was used with the edgeR package to identify differentially expressed genes (DEGs) between symptomatic and asymptomatic IAs. Differential expression was tested using a generalized linear model. Genes with a p-value<0.01 and a fold-change>2 were considered significant. This process was repeated for the PHASES analysis. We further examined biological processes and molecular functions associated with the down and up regulated DEGs for both symptomatic and PHASES analysis using gProfiler, considering those with a p-value<0.05. We also investigated biological significance of the DEGs with Ingenuity Pathway Analysis (IPA) software. Pathways with an |z-score| ≥ 1 were considered significant. Upstream regulators with an |z-score| ≥ 2 were predicted to be inhibited/activated. IPA was also used to construct networks based on gene interactions with the DEGs. A network with a p-score ≥ 21 was considered significant.

We evaluated collinearity between all the significantly different RFs and the normalized gene expression levels of the DEGs for both risk metrics and eliminated all the features that had a high correlation (Pearson correlation coefficient>0.9). To visualize how these features separated high and low-risk IA cases, we performed principal component analysis (PCA) and hierarchical clustering analysis using the final set of non-collinear significantly different RFs alone, DEGs alone, and a combination of both. We first standardized all the RFs and DEGs by removing the mean of each of the features and using unit standard deviation and then computed the principal components and the explained variance of each component using SciKit Learn packages^24^. We then performed hierarchical clustering of the RFs and the DEGs using the online tool Morpheus from Broad Institute. We performed z-score normalization of all the features and used 1-PCC as the metric for hierarchical clustering. Finally, we evaluated potential correlations between the significantly different RFs and DEGs using the Pearson correlation coefficient and the Wald’s test for both risk metrics.

## Results

### Patient Population and Aneurysm Characteristics

Our cohort consisted of 16 patients with IAs of which 3 were symptomatic and 4 had a PHASES score ≥ 6, which we considered high-risk. Through univariate analysis of patient information and aneurysm characteristics, we observed that symptomatic patients were younger, had smaller IAs, and IAs were mostly located in the posterior circulation or at the anterior communicating artery (AComm), although none of these were significantly different. Conversely, we observed that high-risk patients as defined by the PHASES score were older and had larger IAs that were located in the middle cerebral artery and the posterior circulation (Supplementary Table 1). The IAs in the high-risk group also had a statistically significantly higher aspect ratio (p=0.039).

### Radiomics Analysis Between High-risk and Low-risk Aneurysms

A total of 22 RFs were significantly different between symptomatic and asymptomatic IAs (Supplementary Table 2). Of these, 5 were derived from post-contrast MRI, and 17 were from the difference between pre- and post-contrast MRI. We observed that the symptomatic IAs had a lower Gray Level Dependence Matrix (GLDM) large dependence emphasis, Gray Level Run Length Matrix (GLRLM) run variance and Gray Level Size Zone Matrix (GLSZM) large area emphasis in post-contrast MRI. Additionally, they had a higher GLRLM run length non-uniformity, GLSZM zone percentage, and Neighboring Gray Tone Difference Matrix (NGTDM) complexity in the difference between pre- and post-contrast MRI.

In the PHASES based risk assessment, we observed that there were 10 significantly different radiomics features, all of which were based on the difference between pre- and post-contrast MRI. High-risk IAs were characterized by a lower cluster prominence, lower GLSZM small area low grey level emphasis, and a higher GLRLM non-uniformity (Supplementary Table 3) in the difference between post and pre-contrast radiomics. There were no common significant RFs between the two classifications (symptomatic status and PHASES) examined as there were no common cases between both risk metrics.

### Differential Expression Analysis Between High-risk and Low-risk Aneurysms

**Figure 2:**
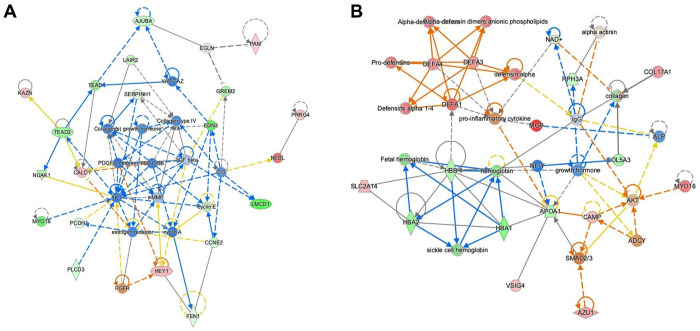
Significant networks from Ingenuity Pathway Analysis. **A)** This top network was constructed using the DEGs identified in the symptomatic analysis. The network reflects dense signaling between collagens, TGF beta, and MMP. **B)** This was the most significant network generated with the DEGs identified in the PHASES analysis. Many signaling pathways in this networks are evident between defensins, cytokines, and the DEGs.

We first examined differential expression for symptomatic vs. asymptomatic IAs. After removing genes with low expression and limiting to protein coding genes, we had a dataset of 13,348 genes. We identified 97 genes that met our criteria for differential expression (p-value<0.01, fold-change>2). Of these, 57 genes had lower expression in the symptomatic group and 40 had increased expression (Supplementary Table 4). No ontologies were associated with the decreased genes, but the genes with increased expression reflected responses to type II interferon, cytokine, peptide, and a defense response to other organism. In our analysis with IPA, macrophage classical activation signaling pathway was the only predicted activated pathway. There were 10 significant upstream regulators, 3 of which were inhibited. Multiple of the activated upstream regulators were cytokines, namely TNF, IFNA2, and IFNB1. In addition, there were 6 significant networks constructed based on connections with the DEG set. The top network reflects signaling centered around TGF-beta, collagens, and MMP as shown in Figure 2A.

In the PHASES analysis, we began with a gene set of 13,177 protein coding genes. Here we identified 38 DEGs, 17 of which had lower expression in the high-risk group and 21 with increased expression (Supplementary Table 5). One gene, *MYO16*, was identified here and in the symptomatic analysis. The down regulated genes were associated with oxygen carrier activity. Biological processes associated with the genes with increased expression in the high-risk group included humoral immune response, cell killing, and organ or tissue specific immune response. Based on these DEGs, IPA identified multiple pathways that were predicted to be activated, which include neutrophil extracellular trap signaling, neutrophil degranulation, and S100 family signaling. No significant upstream regulators were associated with this set of differentially expressed genes. Lastly, there were 2 significant networks. The most significant network had associated functions of cardiovascular disease, cell death and survival, and connective tissue disorders. There were many defensins within this network, along with collagen and pro-inflammatory cytokine as shown in Figure 2B.

### Principal Component and Clustering Analyses

**Figure 3:**
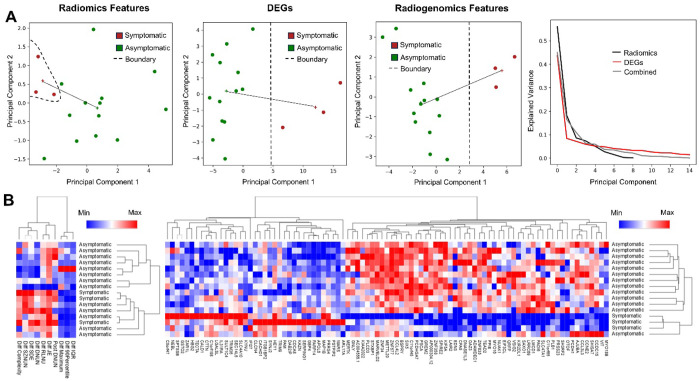
Analysis of symptomatic and asymptomatic IAs. **A)** Principal component analysis of different feature sets (Radiomics, DEGs, and radiogenomics features) based on their symptomatic status. The + represents the cluster centroid and the solid line represents the inter-cluster distance. **B)** Hierarchical clustering of all samples using radiomics and DEGs with respect to their symptomatic status.

A total of 22 RFs and 97 DEGs were used for PCA analysis of symptomatic vs. asymptomatic IAs. We observed that RFs only, DEGs only, and radiogenomics features all had good separation as shown in Figure 3A. We observed that the distance between the centroids of the symptomatic and asymptomatic clusters using RFs only was low (3.61) as compared to PCA performed with DEGs (14.84) and the radiogenomics features (7.07). Through the explained variance, we observed that the elbow point for the DEGs only PCA was the shortest among all feature sets. Through clustering analysis, we observed that 2 of the symptomatic IAs were clustered together using RFs only, whereas all the symptomatic IAs were in proximity while using DEGs only as shown in Figure 3B.

**Figure 4:**
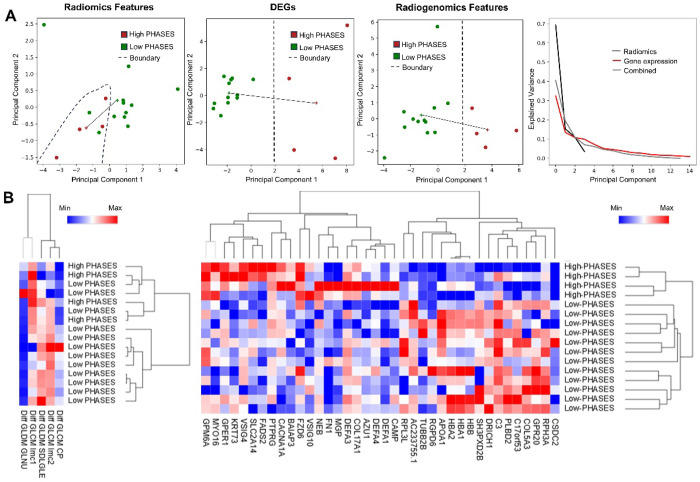
Analysis of high and low-risk IAs based on their PHASES score. **A)** Principal component analysis of different feature sets (Radiomics, DEGs, and radiogenomics features) based on PHASES score. The + represents the cluster centroid and the solid line represents the inter-cluster distance. **B)** Hierarchical clustering of all samples using radiomics and DEGs with respect to their PHASES score (PHASES≥6 was considered high-risk).

In our PHASES based risk assessment, we used 10 RFs and 38 DEGs for PCA analysis. The separation between high-risk and low-risk IAs was moderate using RFs only; however, this improved significantly when using the DEGs and radiogenomics features as shown in Figure 4A. We observed that the distance between the centroids of the high-risk and low-risk clusters using RFs only was low (2.06), as compared to that with DEGs (7.33) and the radiogenomics features (4.98). Similar to symptomatic analysis, the elbow point for the DEGs only PCA was the shortest among all feature sets. Through clustering analysis, we observed that high-risk IAs were separated using RFs only, whereas all the high-risk IAs were clustered together while using DEGs only (Figure 4B).

### Correlation Between Radiomics Features and DEGs

**Figure 5:**
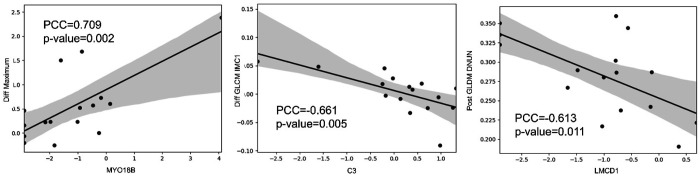
Correlation analysis of significant radiomics features and genes. Correlation plots of representative radiomics features and differentially expressed genes. The light gray region around the solid line represents the 95% confidence interval for the regression plot. **A)** A positive correlation was found between Diff. Maximum and MYO18B expression, while negative correlations were found for **B)** Diff. GLCM IMC1 and C3 expression, and **C)** Post GLDM DNUN and LMCD1 expression.

Through correlation analysis of significantly different RFs and DEGs using symptomatic status, we observed that there were 15 unique RFs that had correlations with 49 DEGs. Approximately 45% of DEGs were correlated to two or more radiomics features. OTOF, MYO18B, and LMCD1 had the most correlates. Of note, we observed that the difference in maximum signal intensity was positively correlated with MYO18B gene (PCC=0.709, p=0.002). We also observed that the GLDM dependence non-uniformity normalized from post-contrast MRI correlates negatively with LMCD1 (PCC=−0.613, p=0.011) and difference in IMC1 between pre- and post-contrast MRI was negatively correlated with C3 (PCC=−0.661, p=0.005) as shown in Figure 5. A complete list of all correlations between RFs and genes is given in Supplementary Table 6. Similarly, correlation analysis of the significantly different RFs and DEGs through PHASES based assessment revealed significant correlations between 5 unique RFs and 6 unique DEGs. However, all of these correlations had a moderate degree of correlation (|PCC|<0.6 and 0.01<p<0.05).

## Discussion

Aneurysm wall enhancement and differential gene expression analysis have individually been demonstrated as tools for robust risk stratification of IAs. In this pilot study, we combined the features obtained from both these modalities and investigated the effects on risk stratification of IAs. We observed that a total of 32 unique radiomics features and 134 unique genes were significantly different between high-risk and low-risk IAs. We also observed that DEGs had the highest variations and inter cluster distances in PCA and grouped well in hierarchical clustering. Finally, we also observed that there were 49 gene correlates to 15 radiomics features. To our knowledge, this is the first study evaluating a combined radiogenomics approach for risk stratification of IAs.

In univariate analysis of RFs, we observed that symptomatic IAs had a lower GLRLM run variance and a lower GLSZM large area emphasis. GLRLM run variance indicates the variance in the run lengths of connected voxels i.e. the heterogeneity in the length of similar intensity voxels. Similarly, GLSZM large area emphasis is the measure of the size of similar intensity regions in the region of interest. A lower value of both RFs in symptomatic IAs can suggest that these have a homogeneous pathobiology, and the IA is in the nascent stage. Similarly, when exploring radiomics differences through PHASES-based risk stratification, we observed high-risk IAs had a lower difference in GLCM cluster prominence, which is indicative of heterogeneity in the IA sac. A lower value in high-risk IAs could mean uniform signal intensity in the tissue potentially indicating uniform enhancement. We also found that high-risk IAs had a higher GLRLM non-uniformity which reflects a higher heterogeneity in post-contrast MRI. Additionally, a lower difference in GLSZM small area low gray level emphasis indicates that post contrast MRI had lower areas of low gray levels. These could be due to focal enhancement of tissue which could result in small patches of high signal intensity in the post-contrast MRI as compared to pre-contrast MRI.

Specifically examining the differentially expressed genes selected in the symptomatic analysis, we note several genes that are involved in critical biological processes, including immune response, cell signaling, and cell differentiation. *ADAMDEC1*, a secreted protein from the disintegrin metalloproteinase family, is associated with immune response, negative regulation of cell adhesions, and proteolysis. *EDN3*, which encodes member of the endothelin family, is a vasoactive peptide that influences an array of biological functions relevant in IA pathogenesis, such as leukocyte chemotaxis, cell proliferation, and smooth muscle contraction. Lastly, *IL31RA*, which encodes a protein belonging to the type I cytokine receptor family, is implicated in immune system processes, including cytokine-mediate signaling, macrophage and monocyte differentiation, and regulation of both apoptotic and proliferative processes. Using IPA to look at broader mechanisms of these differentially expressed genes as a whole, we found interferon signaling, likely in response to activated immune cells, and the macrophage classical activation signaling pathway, which facilitates macrophage-driven inflammation and immune responses. IPA also found TNF signaling, another critical pathway in inflammation and immune responses, to be associated with these genes, suggesting how active these processes are in high-risk IAs.

Focusing on genes identified as differentially expressed between IAs with low and high PHASES score, we again find genes associated with critical processes in IA pathogenesis, such as immune responses, extracellular matrix (ECM) organization, and wound healing. *CAMP* encodes a protein that is crucial for chemotaxis, immune mediator induction, and regulation of the inflammatory response, particularly in chronic inflammation. Further, it plays an active role in defense responses, neutrophil activation, and the positive regulation of angiogenesis and cell proliferation. Defensins, a family of antimicrobial and cytotoxic peptides, are abundant in neutrophil granules and are involved in immune response and chemotaxis. *COL17A1* and *COL5A3* are both key structural constituents of the extracellular matrix, contributing to cell-matrix adhesion and ECM organization. Lastly, *FN1* encodes fibronectin, another key ECM component, supporting angiogenesis, cell-matrix adhesion, and the regulation of cell migration and chemotaxis. The key themes we identified with the complete set of DEGs through IPA include neutrophil degranulation and extracellular traps (NETs), which have been linked to IA rupture^25^. We also note this gene set reflects structural activity, evidenced by collagens and *FN1*, and inflammation, seen through cytokine and NFkB signaling.

From the correlation analysis, we observed that *MYO18B* was significantly correlated with the difference in maximum intensity between the pre- and post-contrast scans. *MYO18B* is a gene that is associated with cell adhesion and migration and had a higher expression in symptomatic IAs in our analysis. A higher degree could lead to disruption in endothelial function thus allowing selective transport of contrast particles into IA wall thus leading to greater enhancement, which is measured by the difference in maximum intensity between pre- and post-contrast MRI. Similarly, we observe a significant correlation between difference of IMC1 and the *C3* gene. The IMC1 measures the correlation of voxels with surrounding voxels with a lower value indicating homogeneous texture. *C3* is associated with inflammatory response and a high homogeneous inflammation could lead to higher differences in IMC1 values. Finally, we also observe a negative correlation of *LMCD1* gene with the dependence non-uniformity normalized (DNUN) from the post-contrast MRI, which is another measure of the homogeneity. *LMCD1* gene is associated with thrombin-induced *IL-33* expression and migration which in turn play a role in endothelial dysfunction^26^. A higher degree of endothelial dysfunction could result in homogeneous proliferation and deposition of contrast particles in the IA wall thus leading to a homogeneous enhancement of the wall.

There are multiple limitations associated with this study. The predominant limitation is the small sample size. It is challenging to identify patients who have undergone VWE and whole blood RNA sequencing. We continue to enroll patients and are collaborating with other centers, so that we can repeat the study in a larger cohort in the future. Another limitation is that symptomatic status and PHASES may not reflect true rupture risk but act as surrogates for this endpoint.

## Conclusion

In this study, we used an established pipeline to assess AWE and genomics analyses to characterize differences between high and low-risk intracranial aneurysms. We observed that a radiogenomics approach by adding genomics to MRI-based radiomics data can help improve risk stratification of IAs. We also observed high degrees of correlation between genomics and radiomics features. These findings demonstrate the utility of radiogenomics in improving risk stratification of IAs.

## Data Availability

The datasets generated or analyzed during the study are available from the corresponding author on reasonable request.

## References

[1] Vlak MHM, Algra A, Brandenburg R, Rinkel GJE. Prevalence of unruptured intracranial aneurysms, with emphasis on sex, age, comorbidity, country, and time period: a systematic review and meta-analysis. The Lancet Neurology. 2011;10:626–636. doi: 10.1016/s1474-4422(11)70109-0 21641282

[2] Wiebers DO, Whisnant JP, Sundt TM, O’Fallon WM. The significance of unruptured intracranial saccular aneurysms. J Neurosurg. 1987;66:23–29. 3783255 10.3171/jns.1987.66.1.0023

[3] Dandurand C, Zhou L, Prakash S, Redekop G, Gooderham P, Haw CS. Cost-effectiveness analysis in patients with an unruptured cerebral aneurysm treated with observation or surgery. J Neurosurg. 2021;135:1608–1616. doi: 10.3171/2020.11.JNS202892 33962376

[4] Froelich JJ, Neilson S, Peters-Wilke J, Dubey A, Thani N, Erasmus A, Carr MW, Hunn AW. Size and Location of Ruptured Intracranial Aneurysms: A 5-Year Clinical Survey. World Neurosurg. 2016;91:260–265. doi: 10.1016/j.wneu.2016.04.044 27108026

[5] Raghuram A, Sanchez S, Wendt L, Cochran S, Ishii D, Osorno C, Bathla G, Koscik TR, Torner J, Hasan D, 3D aneurysm wall enhancement is associated with symptomatic presentation. Journal of NeuroInterventional Surgery. 2022:neurintsurg-2022-019125. doi: 10.1136/jnis-2022-019125 PMC10173164 35853699

[6] Veeturi SS, Saleem A, Ojeda DJ, Sagues E, Sanchez S, Gudino A, Levy EI, Hasan D, Siddiqui AH, Tutino VM, Radiomics-Based Predictive Nomogram for Assessing the Risk of Intracranial Aneurysms. Translational stroke research. 2024. doi: 10.1007/s12975-024-01268-3 PMC12713292 38954365

[7] Ollikainen E, Tulamo R, Frösen J, Lehti S, Honkanen P, Hernesniemi J, Niemelä M, Kovanen PT. Mast Cells, Neovascularization, and Microhemorrhages are Associated With Saccular Intracranial Artery Aneurysm Wall Remodeling. Journal of Neuropathology & Experimental Neurology. 2014;73:855–864. doi: 10.1097/NEN.0000000000000105 25101705

[8] Larsen N, von der Brelie C, Trick D, Riedel CH, Lindner T, Madjidyar J, Jansen O, Synowitz M, Fluh C. Vessel Wall Enhancement in Unruptured Intracranial Aneurysms: An Indicator for Higher Risk of Rupture? High-Resolution MR Imaging and Correlated Histologic Findings. AJNR Am J Neuroradiol. 2018;39:1617–1621. doi: 10.3174/ajnr.A5731 30026386 PMC7655285

[9] Portanova A, Hakakian N, Mikulis DJ, Virmani R, Abdalla WMA, Wasserman BA. Intracranial Vasa Vasorum: Insights and Implications for Imaging. Radiology. 2013;267:667–679. doi: 10.1148/radiol.13112310 23704290

[10] Vergouwen MDI, Backes D, van der Schaaf IC, Hendrikse J, Kleinloog R, Algra A, Rinkel GJE. Gadolinium Enhancement of the Aneurysm Wall in Unruptured Intracranial Aneurysms Is Associated with an Increased Risk of Aneurysm Instability: A Follow-Up Study. AJNR Am J Neuroradiol. 2019;40:1112–1116. doi: 10.3174/ajnr.A6105 31221634 PMC7048551

[11] Larson AS, Lehman VT, Lanzino G, Brinjikji W. Lack of Baseline Intracranial Aneurysm Wall Enhancement Predicts Future Stability: A Systematic Review and Meta-Analysis of Longitudinal Studies. AJNR Am J Neuroradiol. 2020;41:1606–1610. doi: 10.3174/ajnr.A6690 32819901 PMC7583099

[12] Infante T, Cavaliere C, Punzo B, Grimaldi V, Salvatore M, Napoli C. Radiogenomics and Artificial Intelligence Approaches Applied to Cardiac Computed Tomography Angiography and Cardiac Magnetic Resonance for Precision Medicine in Coronary Heart Disease: A Systematic Review. Circ Cardiovasc Imaging. 2021;14:1133–1146. doi: 10.1161/CIRCIMAGING.121.013025 34915726

[13] Liu Z, Duan T, Zhang Y, Weng S, Xu H, Ren Y, Zhang Z, Han X. Radiogenomics: a key component of precision cancer medicine. Br J Cancer. 2023;129:741–753. doi: 10.1038/s41416-023-02317-8 37414827 PMC10449908

[14] Chaddad A, Daniel P, Sabri S, Desrosiers C, Abdulkarim B. Integration of Radiomic and Multi-omic Analyses Predicts Survival of Newly Diagnosed IDH1 Wild-Type Glioblastoma. Cancers (Basel). 2019;11. doi: 10.3390/cancers11081148 PMC6721570 31405148

[15] Zeng H, Chen L, Wang M, Luo Y, Huang Y, Ma X. Integrative radiogenomics analysis for predicting molecular features and survival in clear cell renal cell carcinoma. Aging (Albany NY). 2021;13:9960. 33795526 10.18632/aging.202752 PMC8064160

[16] Poppenberg KE, Chien A, Santo BA, Chaves L, Veeturi SS, Waqas M, Monteiro A, Dmytriw AA, Burkhardt JK, Mokin M, Profiling of Circulating Gene Expression Reveals Molecular Signatures Associated with Intracranial Aneurysm Rupture Risk. Mol Diagn Ther. 2023;27:115–127. doi: 10.1007/s40291-022-00626-x 36460938 PMC9924426

[17] Lindenholz A, van der Kolk AG, Zwanenburg JJM, Hendrikse J. The Use and Pitfalls of Intracranial Vessel Wall Imaging: How We Do It. Radiology. 2018;286:12–28. doi: 10.1148/radiol.2017162096 29261469

[18] Evans RW, Dilli E, Dodick DW. Sentinel headache. Headache. 2009;49:599–603. doi: 10.1111/j.1526-4610.2009.01381.x 19338619

[19] Brinjikji W, Pereira VM, Khumtong R, Kostensky A, Tymianski M, Krings T, Radovanovich I. PHASES and ELAPSS Scores Are Associated with Aneurysm Growth: A Study of 431 Unruptured Intracranial Aneurysms. World Neurosurg. 2018;114:e425–e432. doi: 10.1016/j.wneu.2018.03.003 29530704

[20] Veeturi SS, Pinter NK, Monteiro A, Baig AA, Rai HH, Waqas M, Siddiqui AH, Rajabzadeh-Oghaz H, Tutino VM. An Image-Based Workflow for Objective Vessel Wall Enhancement Quantification in Intracranial Aneurysms. Diagnostics (Basel). 2021;11. doi: 10.3390/diagnostics11101742 PMC8534502 34679440

[21] Veeturi SS, Raghuram A, Miller J, Pinter NK, Sanchez S, Baig AA, Siddiqui AH, Samaniego EA, Tutino VM. Radiomics Features in Contrast-Enhanced and Nonenhanced Magnetic Resonance Imaging Images Are Associated With High Intracranial Aneurysmal Risk. Stroke: Vascular and Interventional Neurology. 2023;3. doi: 10.1161/svin.122.000721 PMC12778691 41583029

[22] van Timmeren JE, Cester D, Tanadini-Lang S, Alkadhi H, Baessler B. Radiomics in medical imaging-“how-to” guide and critical reflection. Insights Imaging. 2020;11:91. doi: 10.1186/s13244-020-00887-2 32785796 PMC7423816

[23] Virtanen P, Gommers R, Oliphant TE, Haberland M, Reddy T, Cournapeau D, Burovski E, Peterson P, Weckesser W, Bright J, SciPy 1.0: fundamental algorithms for scientific computing in Python. Nat Methods. 2020;17:261–272. doi: 10.1038/s41592-019-0686-2 32015543 PMC7056644

[24] Pedregosa F, Varoquaux G, Gramfort A, Michel V, Thirion B, Grisel O, Blondel M, Prettenhofer P, Weiss R, Dubourg V. Scikit-learn: Machine learning in Python. the Journal of machine Learning research. 2011;12:2825–2830.

[25] Korai M, Purcell J, Kamio Y, Mitsui K, Furukawa H, Yokosuka K, Miyamoto T, Sato H, Sato H, Eguchi S, Neutrophil Extracellular Traps Promote the Development of Intracranial Aneurysm Rupture. Hypertension. 2021;77:2084–2093. doi: 10.1161/HYPERTENSIONAHA.120.16252 33813846 PMC8208639

[26] Govatati S, Pichavaram P, Janjanam J, Zhang B, Singh NK, Mani AM, Traylor JG Jr., Orr AW, Rao GN. NFATc1-E2F1-LMCD1-Mediated IL-33 Expression by Thrombin Is Required for Injury-Induced Neointima Formation. Arterioscler Thromb Vasc Biol. 2019;39:1212–1226. doi: 10.1161/ATVBAHA.119.312729 31043075 PMC6540998

